# Rewetting drained boreal peatland forests does not mitigate climate warming in the twenty-first century

**DOI:** 10.1007/s13280-025-02225-6

**Published:** 2025-08-26

**Authors:** Samuli Launiainen, Anssi Ahtikoski, Janne Rinne, Paavo Ojanen, Hannu Hökkä

**Affiliations:** 1 Bioeconomy and Environment, Ecosystems and Modeling, Natural Resources Institute Finland, Latokartanonkaari 9, 00790 Helsinki, Finland; 2 Natural Resources, Forest Management, Natural Resources Institute Finland, Tekniikankatu 1, 33720 Tampere, Finland; 3 Natural Resources, Soil Ecosystems, Natural Resources Institute Finland, Latokartanonkaari 9, 00790 Helsinki, Finland; 4 Natural Resources, Forest Management, Natural Resources Institute Finland, Paavo Havaksen Tie 3, 90570 Oulu, Finland; 5 Department of Forest Sciences, University of Helsinki, Latokartanonkaari 7, 00790 Helsinki, Finland

**Keywords:** Climate change mitigation, Forest peatland restoration, Greenhouse-gas balance, Radiative forcing, Rewetting

## Abstract

Rewetting drained peatland forests restores pristine ecosystem functions, improves peatland ecological status, and has been considered to mitigate climate change. We quantified climate impact of rewetting boreal peatland forests in Northern Europe by comparing the radiative forcing of alternative restoration pathways to that of continued forestry use. We considered changes in soil carbon dioxide, methane and nitrous oxide balance, tree stand carbon sink-source dynamics, albedo change, and included the wood product carbon storage and release. We show that restoring nutrient-rich drained boreal peatland forests contributes to climate warming in the short and medium term (< 200 yr), except in specific cases when tree stand carbon storage is preserved. Rewetting nutrient-poor peatland forests has a persistent warming impact. Our results indicate the ecological benefits of rewetting drained boreal peatland forests come at a climate cost, and that restoration is unlikely to mitigate climate change within a timeframe relevant to the EU climate goals.

**Supplementary Information:**

The online version contains supplementary material available at 10.1007/s13280-025-02225-6.

## Introduction

Restoration of boreal peatlands drained for forestry benefits a multitude of ecosystem services, such as biodiversity and hydrological cycle (Laine et al. 2011; Andersen et al. 2017; Elo et al. 2024; Jurasinski et al. 2024). It is also considered to mitigate climate change (Escobar et al. 2022; Jurasinski et al. 2024), yet some studies challenge this view (Ojanen and Minkkinen 2020). Recently, Laine et al. (2024) evaluated the impact of rewetting on the atmospheric radiative forcing ($\Delta RF$, e.g. Frolking et al. 2006) and proposed that restoring nutrient-rich forest peatlands provide immediate climate benefits. They, however, considered only the change in soil greenhouse gas (GHG) balance following rewetting, provoking the question of how robust the conclusions are if the scope is broadened to include strong carbon (C) sequestration in managed peatland forest stands, forest harvesting and subsequent release of C from created wood products (Jurasinski et al. 2024).

In the Nordic and Baltic countries, ca. 30% of the boreal peatland area has been drained for forestry during the last century (Laine et al. 2009). In Finland, 4.9 Mha (54% of all peatlands) have been drained since the early 1900s (Korhonen et al. 2024), after the pioneering studies of Cajander (1906) and Tanttu (1915) suggested the growth of poorly productive naturally forested peatlands and paludified forests can be greatly improved by drainage. Forestry drainage was strongly expanded in the 1960s and 1970s by the financial support from the state, and a shift from manual to mechanized digging of the ditches. During that time, drainage was conducted also in peatlands that were later found unsuitable for wood production. Drainage of pristine peatlands was ended by 2000. Currently there are 0.6–0.8 Mha of drained peatlands, mostly nutrient-poor bogs, where wood production is not economically feasible (Laiho et al. 2016; Korhonen et al. 2024). In addition, ca. 0.8 Mha of productive drained forest peatlands are reaching the end of their 1st rotation cycle within the next decade, opening a window of opportunity to make smart decisions on their future (Korhonen et al. 2024). Managing for ecological benefits by rewetting and restoration is an option that would comply with the European Nature Restoration Law (European Commission 2022; Hering et al. 2023) but compromise wood production (Jurasinski et al. 2024). Moreover, whether restoring drained forest peatlands is synergetic or acts against reaching climate change mitigation targets of European Climate Law (Kulovesi et al. 2024) remains uncertain, yet decisions are urgent.

Drainage deepened the water table (WT), resulting in a thicker aerobic layer and enhanced peat decomposition and associated carbon dioxide (CO_2_) and nitrous oxide (N_2_O) emissions to the atmosphere (Laine et al. 1996; Ojanen et al. 2013; Minkkinen et al. 2020). At the same time, methane (CH_4_) emissions have decreased (Ojanen et al. 2013), and C accumulation into the growing tree biomass has been rapid (Minkkinen et al., 2001). While peat decomposition has accelerated in drained forest peatlands, accumulation of new carbon into living biomass and topsoil mor humus layer has led to net C sequestration at the ecosystem level (Minkkinen et al. 2002; Lohila et al. 2011; Korkiakoski et al. 2023; Tong et al. 2024). In nutrient-poor forest peatlands also, soil can be a net C sink, similarly to pristine peatlands (Ojanen and Minkkinen 2019; Minkkinen et al. 2020). The positive climate impact of the enhanced C sink after forestry drainage has been partly counteracted by decreased surface albedo (Lohila et al. 2010), but studies are consistent on the net cooling effect on global climate over the first forest rotation period after drainage (Laine et al. 1996; Minkkinen et al. 2002; Lohila et al. 2010).

After successful rewetting, hydrological functions and WT dynamics of undrained peatlands are restored, causing a cascade of biological, ecological, and biogeophysical changes that recover the ecosystem functions of pristine peatlands (Escobar et al. 2022). The peatland GHG balance dynamics after rewetting remain poorly quantified, but the shallower aerobic layer reduces the rate of organic matter decomposition, thereby increasing soil C sequestration or decreasing net CO_2_ emissions. The CH_4_ emissions are known to gradually increase, while N_2_O emissions decrease to a very low level (Minkkinen et al. 2020; Escobar et al. 2022). Overall, studies suggest that GHG balances return to levels comparable with pristine peatlands 15–30 yr after restoration (Laine et al. 2019; Purre et al. 2019; Minkkinen et al. 2020; Escobar et al. 2022).

**Fig. 1:**
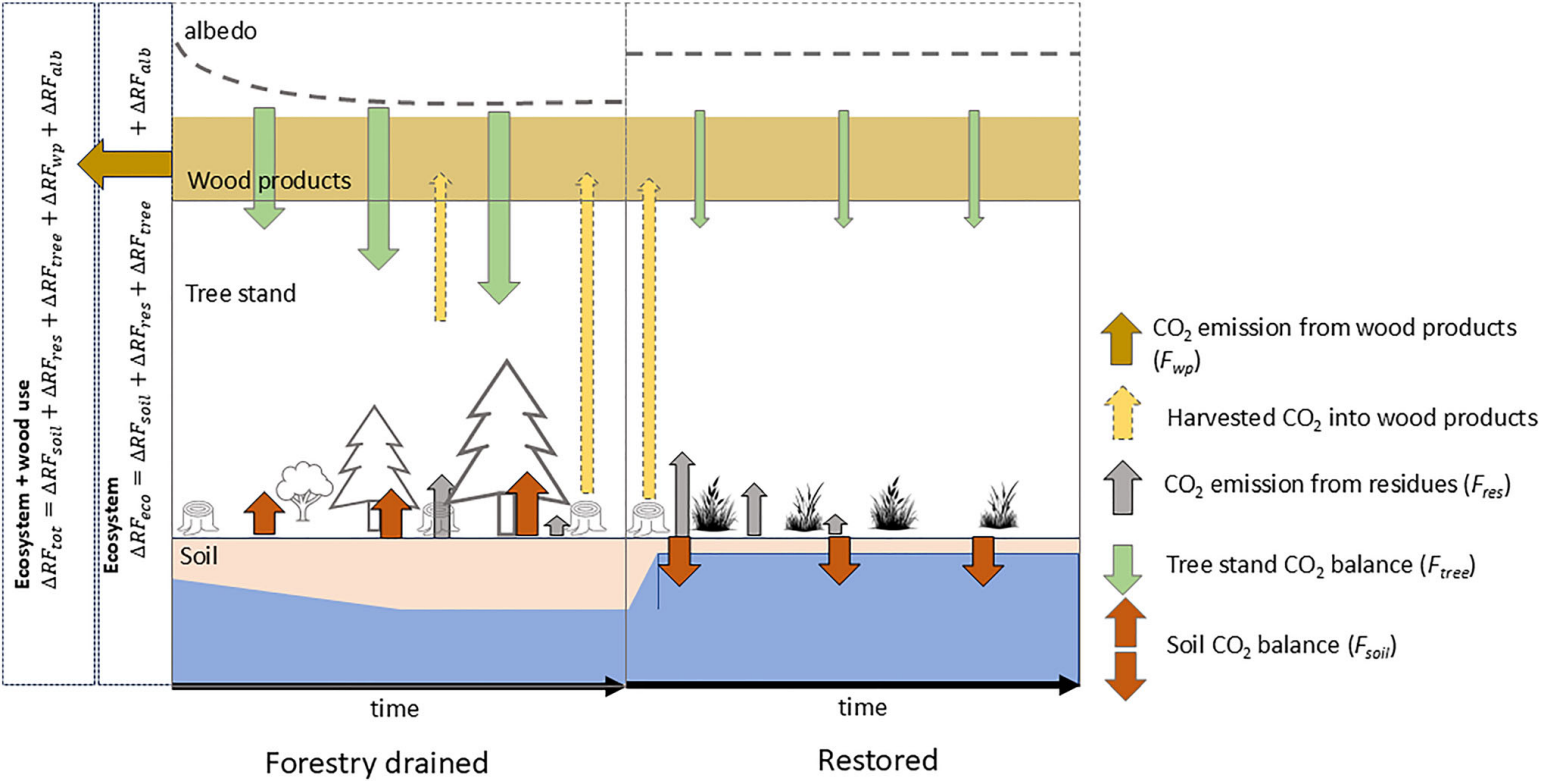
Schematics of carbon dioxide (CO_2_) sinks/sources (i.e., CO_2_ balance) and albedo during a rotation cycle of nutrient-rich drained peatland forest, and the expected situation after restoration to open peatland. The arrow size illustrates flux magnitude. The change in the atmospheric radiative forcing ${\Delta RF}_{\text{tot}}$ summarizes the warming/cooling impact caused by the changes in soil, tree stand, residue and wood product CO_2_ balances between forestry drained and restored. Depending on the system boundaries, release of CO_2_ from wood products is either included or excluded from the analysis

Recently, Laine et al. (2024) defined the plausible restoration outcomes for drained peatland forests in Finland and showed that when nutrient-rich peatland forests are restored, their soil turns from a CO_2_ source to a sink (see Table 1), and the associated cooling is stronger than the warming caused by elevated CH_4_ emissions (i.e. $\Delta RF<0)$. Laine et al. (2024) concluded that restoring nutrient-rich peatlands to forested mires yields immediate climate benefits, while the climate mitigation potential of restoring nutrient-poor peatlands is weak. Their results are, however, conditional to the fact that post-restoration change in only soil GHG balance was accounted for, and transition from drained to restored state assumed instantaneous. Here, we complement their analysis by including the tree stand C sink-source dynamics, approximate the direct radiative forcing of the albedo change, and broaden the system boundaries to include the fate of wood product C storage on $\Delta RF$ (Fig. 1). We show that restoring drained boreal forest peatlands contributes to climate warming in short and medium term (< 200 yr), except in a specific case when tree stand C storage of a on nutrient-rich peatland can be preserved.

**Table 1:** Soil GHG balances (g (gas) m^−2^ a^−1^) used in this study. For CO_2_, the rotation cycle average of Eq. S3 (Fig. S4) and range corresponding to young and mature stands (in parenthesis) are given. The values are from Laine et al. (2024), with exception of drained peatland forests for which they used constant values + 265 gCO_2_ m^−2^ a^−1^ (FNR) and − 45 gCO_2_ m^−2^ a^−1^ (FNP). Data sources and uncertainties are described in Laine et al. (2024)

Peatland type	Soil gas balance (g (gas) m^−2^ a^−1^)		
	CO_2_	CH_4_	N_2_O
Drained nutrient rich (FNR)	+ 384 (140…490)	+ 0.34	+ 0.23
Drained nutrient poor (FNP)	− 15 (− 130…+ 40)	+ 0.34	+ 0.08
Spruce mire	− 91	+ 1.7	+ 0.10
Pine mire	− 97	+ 4.8	+ 0.03
Open eu/mesotrophic	− 104	+ 15	+ 0.10
Open oligotrophic	− 124	+ 22	+ 0.03
Open ombotrophic	− 95	+ 9.7	+ 0.03

## Materials and methods

### Hypothetical restoration pathways

We illustrate the effects of dynamic tree stand CO_2_ sink, the fate of harvested wood products, and the albedo change on $\Delta RF$ using hypothetical restoration cases:

- *Case 1*: Restoration of a nutrient-rich forest peatland (FNR) in Southern Finland to an open eutrophic/mesotrophic peatland. We assume restoration takes place via clear-cutting a mature tree stand, which the stem wood is allocated to short- and long-term wood products. Harvest residues are left to decompose on-site. The restoration impact on soil CO_2_, CH_4_ and N_2_O fluxes (${F}_{k,\text{soil}}$) and albedo are assumed to be instantaneous. In the restoration scenario, wood product and residue C pools are depleted over time, while in the reference forestry scenario they are periodically replenished through harvests.
- *Case 2*: Restoration of FNR to a spruce mire, assuming no harvest is conducted, and tree stand C storage is preserved after restoration. According to Laine et al. (2024), *Case 1*&*2* restoration pathways offer the strongest climate benefits. This raises the question: to what extent does accounting for the tree stand C sequestration, harvests and wood use alter their conclusion?
- *Case 3*: Assessment of how the climate impact depends on timing of restoration? Building on *Case 1*, Restoration is now initiated at different points during the 58 yr forest rotation cycle.
- *Case 4*: Evaluation of how a gradual rather than instantaneous change of ${F}_{k,\text{soil}}$ from drained to restored state affects $\Delta \text{RF}$? We assume net soil GHG balances change linearly over a post-restoration period (${\tau }_{r}$) up to 40 yr, covering the typical equilibration time of 15–30 yr (Escobar et al. 2022). Finally, we compare our results to those of Laine et al. (2024).

In all cases, the reference scenario is even-aged forest management, where rotation cycles and management practices continue unchanged for the next 200 years.

### Estimating the change in net GHG fluxes and atmospheric radiative forcing

We adopt, as far as possible, the same assumptions and parameters as Laine et al. (2024). Detailed description of the methods, underlying assumptions and data used in this study are provided in the Supplementary Material (Suppl.).

A simple book-keeping model is used to track changes in C storage (${S}_{i}(t)$,  C m^−2^) over time (*t*) in soil, tree stand, harvest residues, and wood products made from harvested biomass (Suppl. S1). The model yields the annual net flux of CO_2_ between the atmosphere and the peatland–wood product system ${F}_{\text{co}2}(t)$ (g CO_2_ m^−2^ a^−1^):

1 $$ F_{{{\text{co2}}}} \left( t \right) = F_{{\text{co2,soil}}} \left( t \right) - F_{{{\text{tree}}}} \left( t \right) + F_{{{\text{res}}}} \left( t \right) + F_{{{\text{wp}}}} \left( t \right), $$

where negative ${F}_{\text{co}2}$ indicates net CO_2_ uptake. The soil CO_2_ balance (${F}_{\text{co}2,\text{soil}}$), tree stand biomass change (${F}_{\text{tree}}$), and CO_2_ emissions from residue decomposition (${F}_{\text{res}}$) sum up to the net ecosystem exchange (NEE). The full Eq. 1 also includes CO_2_ emissions from wood products (${F}_{\text{wp}})$, thus accounting for the dynamics of the wood product C storage.

Biomass increment and ${F}_{\text{tree}}(t)$ are simulated using the Motti forest simulator (Hynynen et al. 2005) following the current guidelines for even-aged forestry in Finland (Kellomäki 2022). Forest dynamics are modeled across a range of site types representing peatland forests ranging from eutrophy to oligo-ombrotrophy, and for climate conditions in Southern and Northern Finland (Suppl. S1.5). The water table deepens with increasing stem volume (Vol, m^3^ ha^−1^) based on Sarkkola et al. (2010), which influences the soil CO_2_ balance following Ojanen and Minkkinen (2019). Our formulation is a dynamic version of that used in Laine et al. (2024) to estimate drained peatland forest soil CO_2_ balance (Table 1, Fig. S4).

During the rotation period, biomass is removed through thinnings (partial harvests) and final clear-cut. Harvests provide input to harvest residue pools that decompose on-site, and to short- (mean lifetime τ = 3 yr, incl. bioenergy) and long-term (τ = 30 yr) wood product pools. These pools emit CO_2_ at rates proportional to their size and decay rate: ${F}_{\text{wi}}(t)={S}_{\text{wi}}(t){e}^{-1/{\tau }_{i}}$, where ${\tau }_{i}$= 3–300 yr is the mean lifetime of pool *i*. For restored peatlands, we assume that stumps and roots decompose slowly (τ = 300 yr) in anoxic conditions, ${F}_{\text{tree}}\left(t\right)$= 0 and ${F}_{\text{co}2,\text{soil}}$ is constant in time. For CH_4_ and N_2_O assume that only soil and forest floor processes contribute to their balances that remain constant over time but differ between peatland types and between drained vs. restored scenarios (Table 1). Thus, for CH_4_ and N_2_O, Eq. 1 reduces to ${F}_{\text{ch}4}={F}_{\text{ch}4,\text{soil}}$ and ${F}_{\text{n}2\text{o}}={F}_{\text{n}2\text{o},\text{soil}}$.

Impact of restoration on GHG balance is computed as the difference between restored (*r*) and drained (*d*) peatland, i.e., ${\Delta F}_{\text{co}2, d\to r}\left(t\right)={F}_{r}\left(t\right)- {F}_{d}\left(t\right)$. The REFUGE 4 method (Lindroos 2023) is used to quantify how such change in the net CO_2_, CH_4_ and N_2_O uptake/emission affects their atmospheric stocks and radiative forcing (Suppl. S1.3). The approach accounts for the dynamic response of atmospheric GHG storages to surface emissions/sinks and includes the effects of atmospheric chemistry and land–ocean GHG sink.

The change in annual radiative forcing ${\Delta RF}_{k}(t)$ (W m^−2^ (earth) m^−2^ (land restored)) of gas *k* contributes either to climate warming $({\Delta RF}_{k}>0)$ or cooling (${\Delta RF}_{k}<0)$. The radiative forcings are additive, and the time-dependent total radiative forcing from forest peatland restoration is:

2 $$ \begin{aligned} \Delta RF_{{{\text{tot}}}} \left( t \right) & = \Delta RF_{{\text{co2,soil}}} \left( t \right) + \Delta RF_{{{\text{tree}}}} \left( t \right) + \Delta RF_{{{\text{res}}}} \left( t \right) + \Delta RF_{{{\text{wp}}}} \left( t \right) \\ & \quad + \Delta RF_{{{\text{ch4}}}} \left( t \right) + \Delta RF_{{{\text{n2o}}}} \left( t \right) + \Delta RF_{{{\text{alb}}}} \left( t \right), \\ \end{aligned} $$

where the last term ${\Delta RF}_{alb}$ approximates the direct radiative forcing due to change in the surface albedo (Suppl. S1.4). Equations 1 and 2 enable analyzing how the dynamic changes in different GHG fluxes and (eco)system components contribute to ${\Delta RF}_{\text{tot}}\left(t\right)$.

## Results

**Fig. 2:**
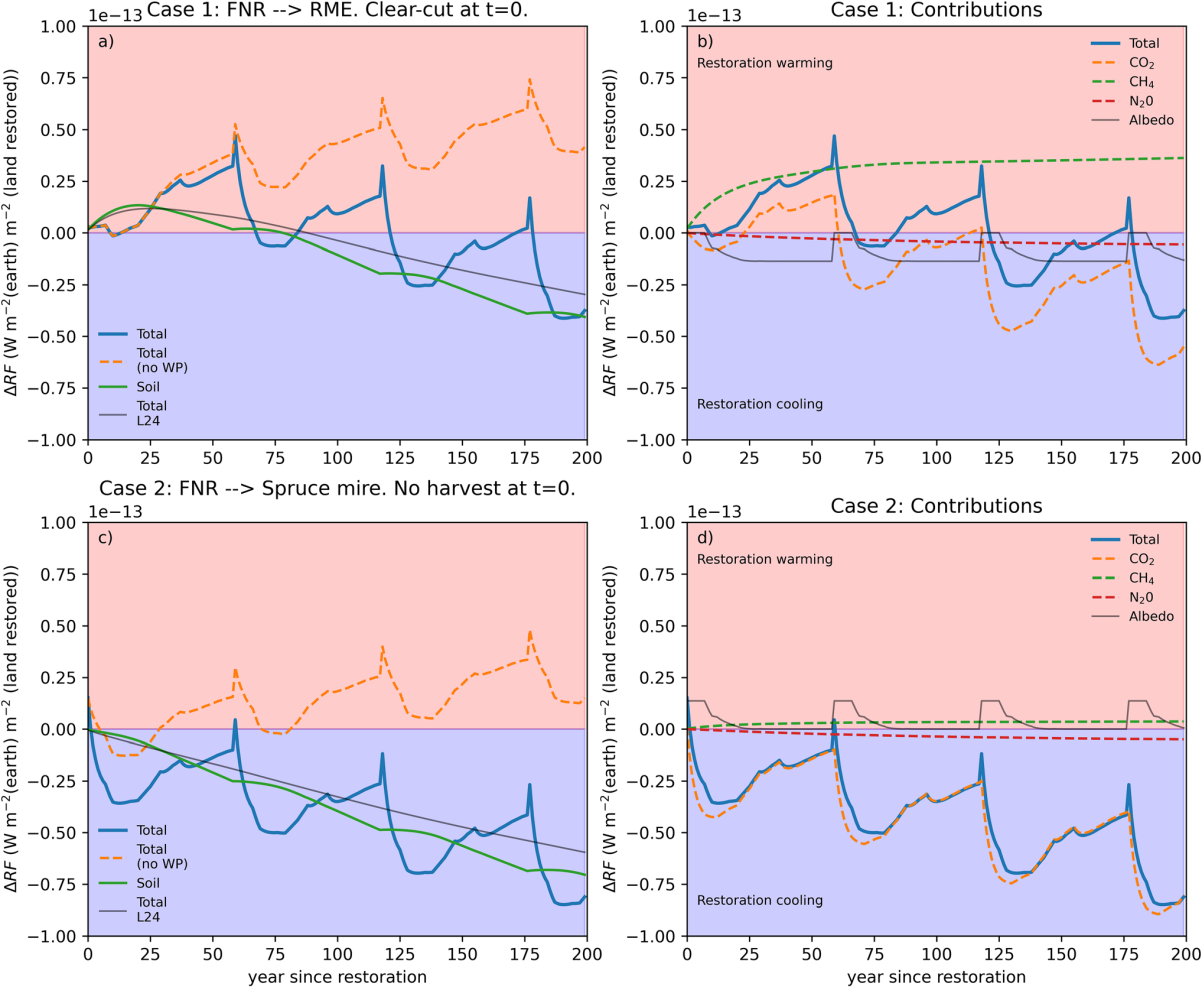
Change in the annual radiative forcing ${\Delta RF}_{\text{tot}}$ (**a**) and its components (**b**). *Case 1:* Restoring nutrient-rich forest (FNR, mesotrophic Mtkg in Southern Finland) to an open eutrophic/mesotrophic peatland by clear-cutting at the end of rotation period. Development of C storages and CO_2_ fluxes between the considered system and the atmosphere are shown in Fig. S1. *Case 2* (**c**, **d**) show ${\Delta RF}_{tot}$ when the same forest is restored to a tree-covered mire leaving the tree stand intact, assuming it preserves its C storage infinitely. In left panels (**a**, **c**) the thin black line (L24) shows the estimates of Laine et al. (2024), which include only on the change in soil GHG balances. Orange dashed line (no WP) shows ${\Delta RF}_{\text{tot}}$ if the release of CO_2_ from the wood products is not accounted for. Figure S3 shows the impact of forest productivity on *Case 1*

In *Case 1*, a fertile spruce stand at the end of its rotation (age 58 yr, Vol. ~ 400 m^3^ ha^−1^, mean annual increment in late rotation ~ 10 m^3^ ha^−1^ a^−1^; Fig. S1 and 2) on a mesotrophic (Mtkg) drained peatland in Southern Finland is restored to an open eutrophic/mesotrophic fen (Fig. 2a, b). Over the forest rotation, 28% of the harvested stem wood was allocated to long-term forest products. Restoration contributes to climate warming (${\Delta RF}_{\text{tot}}$>0) over the first 58 yr forest rotation period and for most of the 2nd rotation cycle. Restoration starts to provide continuous climate benefits (${\Delta RF}_{\text{tot}}$<0) only after the third rotation, but the average contribution remains warming for ca. 200 yr (Figs. 2a, b and 4a). Stand productivity has significant impact on ${\Delta RF}_{\text{tot}}$<0: the more productive the restored NRF stands are, the stronger and more long-term the associated warming impact (Fig. S3).

The role of different (eco)system components and GHGs on ${\Delta RF}_{\text{tot}}$ varies over time, as their contributions are affected by stand, residue and wood product dynamics impact on net CO_2_ source/sink strength (Eq. 1; Fig. S1), and the differing atmospheric lifetimes of GHGs (Suppl. S1.3). Increasing surface albedo after restoration creates a persistent cooling effect, which is the strongest when compared to mature forests (Fig. 2b and Fig. S4). Soon after restoration, increasing methane emissions have a major warming impact but the effect saturates due to the short atmospheric lifetime of CH_4_ (Frolking et al. 2006). Increasing CH_4_ emissions also explain why ${\Delta RF}_{\text{soil}}$ is positive for the first ca. 60–80 yrs after rewetting. Thereafter the role of CO_2_ becomes more prominent and the change in soil GHG balance creates a cooling effect because. This occurs because the restored peatland soil is assumed to be a constant sink of CO_2_ (Table 1), while net emissions from drained forest peatland soil increase with deepening of WT toward the end of rotation (Suppl. S1.1; Fig. S4). After the first decades, the variability in ${\Delta RF}_{\text{tot}}$ is driven by the cyclic C sequestration and release from the ecosystem components (on-site) and wood products (Fig. 2a and Fig. S1). The C sequestered into tree biomass is converted into residues and wood products after partial harvests (thinnings) and the final clear-cut at the end of rotation, resulting in a characteristic ‘saw-tooth’ pattern in ${\Delta RF}_{\text{tot}}$ (Fig. 2a). At the end of rotation period, the forest stand CO_2_ sink is temporarily removed, and rapid release of C stored in residues and wood products yields strong net CO_2_ emissions (Fig. S1) into the atmosphere, causing the drop in ${\Delta RF}_{\text{tot}}$.

On timescales longer than rotation period, the net C sequestration into living biomass, residues or wood products is small, and the long-term trend in ${\Delta RF}_{\text{tot}}$ is driven by the difference of soil C storage development between restoration (increase) and forestry (decrease in FNR, increase in FNP) scenarios (Table 1, Fig. S1). This also explains the overall cooling contribution of CO_2_ (Fig. 2b). Thus, the centennial-scale dynamics of ${\Delta RF}_{\text{tot}}$ caused by restoring into an open peatland is driven mainly by the change in soil GHG balance, as implicitly assumed in Laine et al. (2024). However, the estimated near-future climate impacts are strikingly different depending on whether only the soil or the entire system GHG balance is considered.

In *Case 2,* the same forest is restored into a spruce mire now leaving tree stand intact, and assuming it preserves its C storage ad infinitum (Fig. 2c). This restoration pathway provides an immediate and persistent cooling effect (${\Delta RF}_{\text{tot}}$<0), mainly because the initial CO_2_ emissions from wood products and harvest residues are avoided in the restoration scenario. Also, the increase of CH_4_ emissions from drained to restored state is smaller than in *Case 1* (Table 1) and resulting warming impact (${\Delta RF}_{\text{ch}4}$>0) remains small, and total climate impact is driven by CO_2_ (Fig. 2d). The effect of *Case 2* rewetting on ${\Delta RF}_{\text{alb}}$ is opposite to that of *Case 1*, as albedo of mature (restored) forest stand is lower than in young managed stands. The difference between *Case 1* and *2* demonstrates how central the fate of pre-restoration tree stand C storage is for the climate impact.

**Fig. 3:**
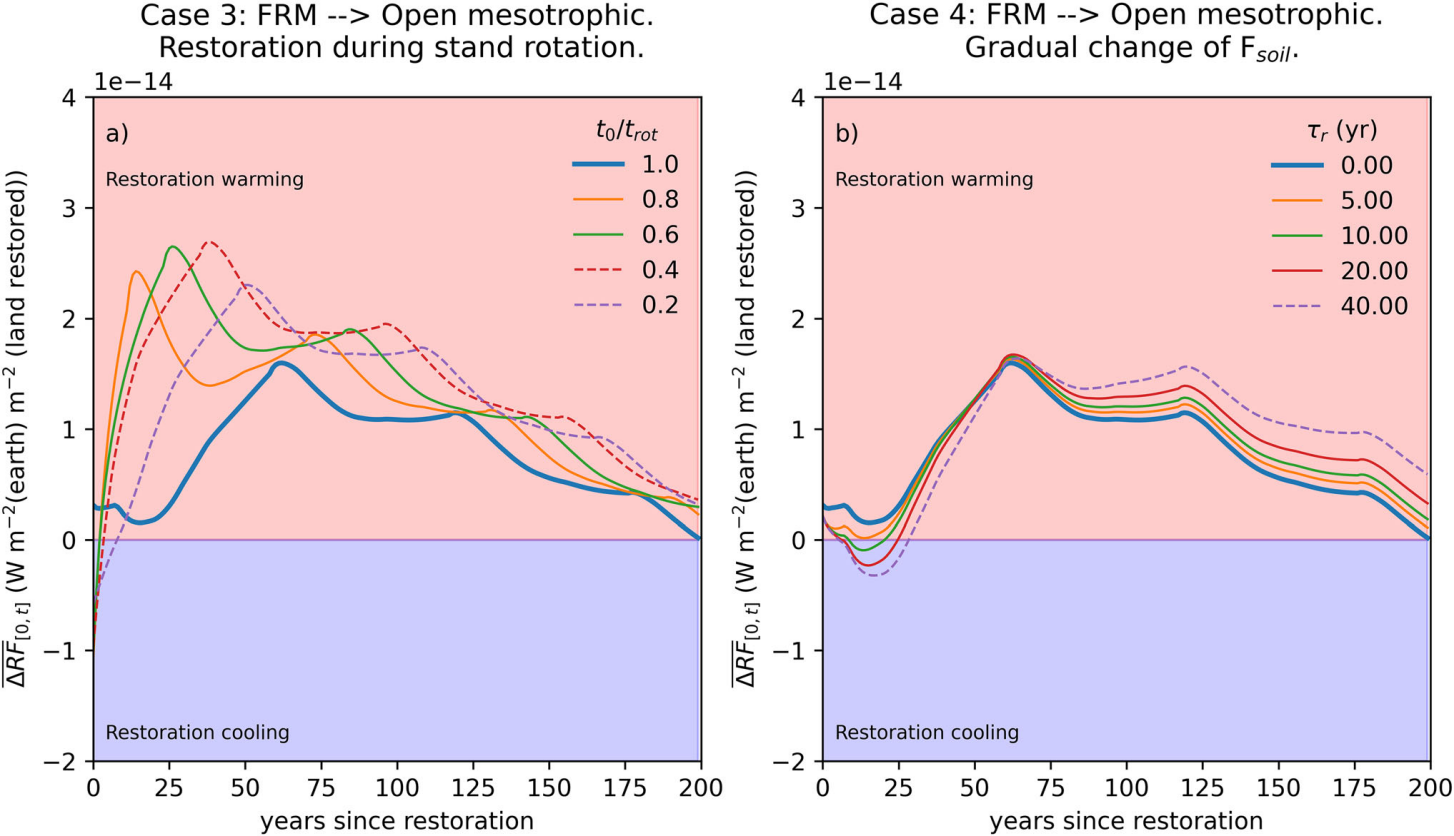
Effect of conducting *Case 1* restoration during the forest rotation cycle (**a**). The lines show average change in total radiative forcing (${\overline{\Delta RF}}_{[0, t]}$) from time t = 0 until a given point in time (in x-axis). Restoration during the forest rotation (${t}_{0}/{t}_{\text{rot}}$ < 1) leads to stronger short- and long-term warming than rewetting at the rotation end. A gradual change in soil GHG balance from drained to restored state over a period ${\tau }_{r}$ (**b**) has only a minor impact on ${\overline{\Delta RF}}_{[0, t]}.$ Thick blue line is same in both panels and equals time-averaged ${\Delta RF}_{\text{tot}}$ from Fig. 2a

In previous cases, restoration was done at the end of rotation in tandem with clear-cutting and regeneration (Fig. 2). In real world, rewetting a peatland area requires restoration measures are applied simultaneously at different-aged stands. In *Case 3*, we initiate *Case 1* restoration at different times during the 58 yr rotation cycle (Fig. 3a). The results show interesting dynamics with respect to timing of restoration relative to the rotation length of managed forest. The caused long-term warming impact is the stronger the younger the restored stands are, as the C sink of an established, well-growing tree stand is lost for the remaining rotation period. On the other hand, the most unfavorable short-term climate impact occurs when mature forest stands are restored, as the earlier loss of biomass C storage leads to earlier large CO_2_ emissions to the atmosphere compared to continued forestry scenario. When the alternative, as in this analysis, is to continue fixed-length rotation forestry, restoration to open peatland habitats causes least climate harm if it can be done at end of rotation cycle. On peatland scale this is, however, rarely practical.

The gradual rather than instantaneous transition of the soil GHG balances from drained to restored state (*Case 4*, Fig. 3b) has only a minor effect on ${\Delta RF}_{\text{tot}}$, which pales in comparison to timing of restoration (Fig. 3a) and the selected restoration pathway (Fig. 2). This suggests that uncertainty in post-restoration GHG balance equilibration time (Escobar et al. 2022) may not be critical for assessing climate impact dynamics. However, delayed return (${\tau }_{r}$= 40 yr) of pristine ecosystem functions (e.g. gradual increase in CH_4_ emissions) appears to lead to more favorable short-term (< 30 yr) and more negative long-term climate impact compared to the instantaneous $({\tau }_{r}$= 0) GHG balance recovery (Fig. 3b).

## Discussion

**Fig. 4:**
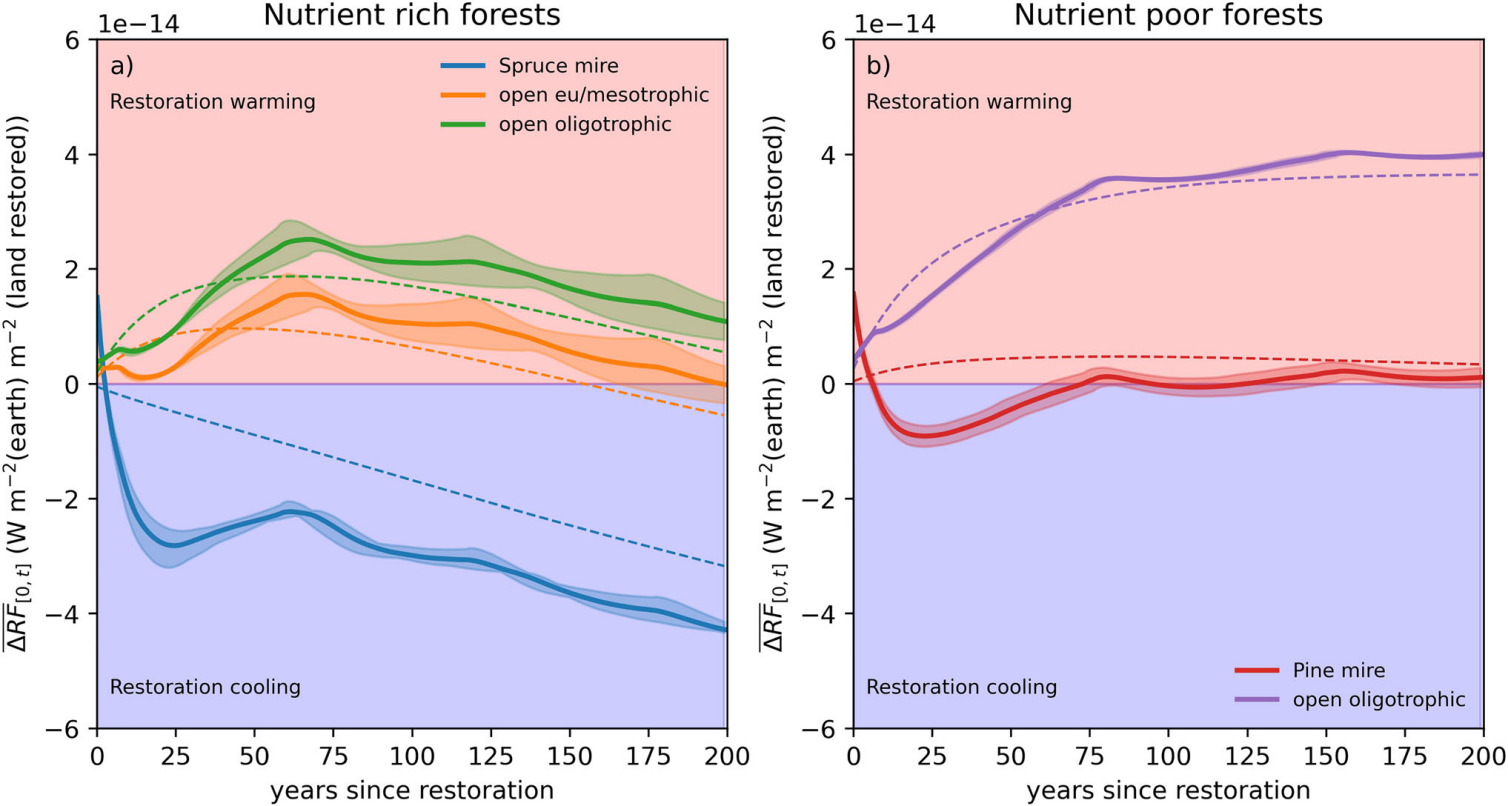
Change in the total radiative forcing (${\Delta RF}_{\text{tot}}$) when a nutrient-rich (**a**) and a nutrient-poor (**b**) drained peatland forest is restored to different habitats. The continuous lines show the average radiative forcing (${\overline{\Delta RF}}_{[0, t]}$) from t = 0 until a given point in time (in x-axis). The colored range shows the variability due to different forest dynamics across site types and south-north climate gradient (see Suppl. S1.5). Vegetation C storage is assumed intact when restoring to tree-covered mires. Dashed lines show comparison to Laine et al. (2024), who considered only the change of soil GHG balances

Rewetting drained boreal forest peatlands is unlikely to mitigate climate change in the twenty-first century. The results unequivocally show that restoring drained forest peatlands to open peatland habitats (Figs. 2a and 4) will contribute to climate warming (${\Delta RF}_{\text{tot}}$> 0) both on short and medium term (< 200 yr), while longer-term benefits may emerge when restoring nutrient-rich sites. Our results align with those by Ojanen and Minkkinen (2020), who showed that restoring boreal forestry drained peatlands will have a warming effect at least for the first century after restoration, depending on forestry practices applied. Although Laine et al. (2024) only considered the impacts of restoration on the soil GHG balance, their results provide similar conclusion (Fig. 4). Our findings are also consistent with earlier studies showing that draining boreal peatlands for forestry has contributed to climate cooling, as C accumulation in the growing tree stand has outweighed C losses from peat soil (Laine et al. 1996; Minkkinen 1999; Minkkinen et al. 2002) and negative effects of decreased albedo (Lohila et al. 2010).

Short- to medium-term (< 200 yr) climate impact of restoration is dictated by the fate of the C sequestered in the tree stand (Figs. 2 and 3a). If the tree stand C storage can be preserved when restoring to tree-covered mires (Figs. 2b and 4), the avoided CO_2_ emissions from decomposing residues and wood products provide climate benefits and it is possible to achieve the anticipated synergies between improved biodiversity and climate mitigation goals (Bullock et al. 2011; Dinesen et al. 2021; Laine et al. 2024). Our results reveal that in an optimal case, successful restoration of nutrient-rich forest peatlands to tree-covered mires (Fig. 4) may provide climate mitigation exceeding that offered by improved soil GHG balance only. However, our analysis also suggest that climate impacts of restoration are highly dependent on the selected restoration targets (stand age, productivity, site type) and desired outcomes (post-restoration habitats), leading to varying synergies and trade-offs between different ecosystem services (Ojanen and Minkkinen 2020; Elo et al. 2024; Laine et al. 2024). For instance, when the restoration targets are open peatland habitats, the adverse short- and medium-term climate warming impact can, to some degree, lessened if restoration is applied to mature instead of young stands (Fig. 3a). Among nutrient-rich forest peatlands, it is less harmful to restore low- than high-productivity stands (compare Fig. 2a,b and Fig. S3).

The fate of the tree stand C storage and sink, and the release of CO_2_ from residues and wood products determine the radiative forcing dynamics at short timescales but for periods significantly longer than the stand rotation, ${\Delta RF}_{\text{tot}}$ trend depends mainly on how the soil C storage develops after restoration compared to that of continued forestry use (Fig. 2a, c). This is because the C storage of wood products and residues is mostly depleted during the forest rotation cycles, the radiative forcing caused by N_2_O emissions is small overall, and that from elevated methane emissions saturates after ca. 100 yrs (Fig. 2b, d). Conclusions on the climate impact of rewetting, and the underlying causal mechanisms are thus highly dependent on the timescale of interest. Focusing on the change in soil GHG balance (Laine et al. 2024) is viable when long-term climate impacts of restoration to open peatlands are considered but gives a biased view in the short-term, and particularly when restoring to tree-covered mires (Fig. 4).

Our analysis also illustrates how the conclusion on the climate impact can differ depending on whether the wood end-use is included (Fig. 2a, c and Fig. S3 blue line) or excluded (dashed orange line). The latter assumption is implicitly made if ${\Delta RF}_{\text{tot}}$ is evaluated at the site level using ecosystem NEE (Fig. 1 and S1). In managed forests this would mean the harvested wood C transported from the site and turned into wood products is omitted from the analysis (or assumed to form an infinite C storage). For timescales longer than the wood product life cycle this is conceptually incorrect and would unrealistically favor the forest management scenario. On the other hand, rewetting a peatland is unlikely to affect regional wood demand in the short term, and restoration may lead to compensatory harvesting elsewhere (harvest leakage; Kallio and Solberg 2018; Schwarze et al. 2002). This means the positive effects of preserving stand C storage when restoring to tree-covered mires (Fig. 2c) would be counteracted by emissions from residues and wood products caused by increased harvests elsewhere. In a broader context, this means that unless restoration affects wood demand, rewetting nutrient-rich peatland forests to tree-covered mires is likely to provide only long-term climate mitigation, analogously to restoring to open peatlands (Figs. 2 and 4). Deeper exploration on roles of system boundaries is beyond the scope of this work, but results highlight the need to consider restoration gains and trade-offs as part of a wider analysis and valuation of the ecologically, environmentally, climatically, and economically sustainable boundaries for using forests and peatlands (e.g. Bullock et al. 2011; Koskinen et al. 2017; Juutinen et al. 2020; Makrickas et al. 2023).

Our model of C storage and GHG balance development over forest rotation cycles assumes that forest management will continue as in the past, omitting potential benefits of a changing environment, altered biogeochemistry and improved management on growth and C sequestration of drained peatlands (Hökkä et al. 2024a,b). The magnitude of the predicted NEE after clear-cutting of a fertile forest peatland is in line with recent observations (Korkiakoski et al. 2023; Tikkasalo et al. 2025), but as we exclude ground vegetation and pioneering vegetation net primary productivity, the recovery of CO_2_ sink after clear-cutting is delayed compared to observations from a fertile drained peatland (Korkiakoski et al. 2023) and from young mineral soil stands (Grelle et al. 2023). Otherwise, NEE dynamics with stand age are realistic compared to those observed in managed boreal forests (Goulden et al. 2011; Peichl et al. 2023). We also omitted the possibility to adapt peatland forestry, e.g., via continuous cover forestry (Nieminen et al. 2018), raising water table for better growth (Hökkä et al. 2024b) and reduced CO_2_ emissions (Ojanen et al. 2013), or by lengthening rotation cycles for improved tree stand C storage. We also neglected the possible changes in wood use and ignored the substitution effects. It can thus be argued that our results may unrealistically favor restoration, as future forest management on peatlands can be adjusted to improve its impact on the climate.

We compared the atmospheric radiative forcing of alternative restoration outcomes to that of continued even-aged forest management. By doing so, we assume forest growth, management, and wood use, as well as restored peatland GHG balance, will remain as in the past for the next 200 years. In absence of post-rewetting data on stand development, we also made the naïve assumption that tree stand C storage is preserved permanently when restoring to tree-covered mires (*Case 2*, Fig. 2c, d). These simplifications mean potential effects of increased abiotic (drought, floods, windthrows, peat fires) and biotic disturbances on peatland forests’ C cycle (Turetsky et al. 2004; Lindner et al. 2014; Venäläinen et al. 2020), restoration success (Elo et al. 2024) and any changes in peatland ecosystem functions that would affect their GHG balance in a future climate (Frolking et al. 2011; Wu and Roulet 2014) are not accounted for. Our analysis focuses on the change in global atmospheric radiative forcing (${\Delta RF}_{\text{tot}}$) and does not consider the biophysical impacts of rewetting on the local surface energy partitioning (Helbig et al. 2020). It has, e.g., been suggested that extensive rewetting of boreal peatlands can buffer against high summer temperatures on a regional scale (Helbig et al. 2020).

## Conclusion and implications

To comply with the European Nature Restoration Law (Hering et al. 2023), the demand to restore drained boreal forest peatlands will increase in the next decade. With limited knowledge and data on post-restoration GHG balances (see review in Escobar et al. 2022), tree growth and restoration success (Elo et al. 2024), and future peatland forest management (Hökkä et al. 2024a,b), predictions of the resulting climate impacts are well-aimed shots into the dark. Still, the objective use of ecosystems ecology of managed forests and natural peatlands is our best asset to inform decision-making on restoration today. Our results, supported by those of Ojanen and Minkkinen (2020) and Laine et al. (2024) show that the ecological benefits of restoring drained boreal peatland forests in the Northern Europe will in most cases have a climate cost (warming impact) throughout the twenty-first century, acting against reaching the EU climate-neutrality 2050 target.

Our results have four key implications for planning restoration of boreal drained peatland forests: (1) Rewetting nutrient-rich production forests to open peatland habitats will contribute to climate warming in the short and medium term (< 200 yr), while restoring nutrient-poor forests leads to even more long-term warming; (2) The adverse climate impact of restoration can be partly mitigated by focusing restoration activities to late-rotation stands; (3) Successful rewetting of nutrient-rich drained peatlands to tree-covered mires can have a cooling effect if the tree stand carbon storage can be preserved; and (4) In most cases, there is a clear trade-off between restoring peatland ecological functions and biodiversity (Elo et al. 2024; Jurasinski et al. 2024) and the lost climate change mitigation (this study). Therefore, it is imperative to consider restoration as part of a broader analysis and valuation of the ecological, environmental, climatic, and economic sustainability boundaries for the use of forests and peatlands.

## Supplementary Information

Below is the link to the electronic supplementary material.

Supplementary file1 (PDF 1143 KB)

## References

[CR1] Andersen, R., C. Farrell, M. Graf, F. Muller, E. Calvar, P. Frankard, S. Caporn, and P. Anderson. 2017. An overview of the progress and challenges of peatland restoration in Western Europe. *Restoration Ecology* 25: 271–282.

[CR2] Bullock, J. M., J. Aronson, A. C. Newton, R. F. Pywell, and J. M. Rey-Benayas. 2011. Restoration of ecosystem services and biodiversity: Conflicts and opportunities. *Trends in Ecology & Evolution* 26: 541–549. 21782273 10.1016/j.tree.2011.06.011

[CR3] Cajander, A. K. 1906. Studien uber die Moore Finnlands. *Acta Forestalia Fennica* 2: 1–208.

[CR4] Højgård Petersen, A., and C. Rahbek. 2021. Synergy in conservation of biodiversity and climate change mitigation: Nordic peatlands and forests. *Nordic Council of Ministers*, 2021: 510 (report).

[CR5] Elo, M., S. Kareksela, O. Ovaskainen, N. Abrego, J. Niku, S. Taskinen, K. Aapala, and J. S. Kotiaho. 2024. Restoration of forestry-drained boreal peatland ecosystems can effectively stop and reverse ecosystem degradation. *Communications Earth & Environment* 5: 680. 39610898 10.1038/s43247-024-01844-3 PMC11599035

[CR6] Escobar, D., S. Belyazid, and S. Manzoni. 2022. Back to the future: Restoring northern drained forested peatlands for climate change mitigation. *Frontiers in Environmental Science* 10: 834371.

[CR7] European Commission, 2022. Nature Restoration Law—For People, Climate, and Planet. https://op.europa.eu/publication-detail/-/publication/a0e3cfac-f600-11ec-b976-01aa75ed71a1/language-en

[CR8] Frolking, S., N. Roulet, and J. Fuglestvedt. 2006. How northern peatlands influence the Earth’s radiative budget: Sustained methane emissions versus sustained carbon sequestration. *Journal of Geophysical Research* 111: 2006. 10.1029/2005JG000091.

[CR9] Frolking, S., J. Talbot, M. C. Jones, C. C. Treat, J. B. Kauffman, E. S. Tuittila, and N. Roulet. 2011. Peatlands in the Earth’s 21st century climate system. *Environmental Reviews* 19: 371–396.

[CR10] Goulden, M. L., A. McMillan, G. Winston, A. Rocha, K. Manies, J. W. Harden, and B. Bond-Lamberty. 2011. Patterns of NPP, GPP, respiration, and NEP during boreal forest succession. *Global Change Biology* 17: 855–871.

[CR11] Grelle, A., P. O. Hedwall, M. Strömgren, C. Håkansson, and J. Bergh. 2023. From source to sink–recovery of the carbon balance in young forests. *Agricultural and Forest Meteorology* 330: 109290.

[CR12] Helbig, M., J. M. Waddington, P. Alekseychik, B. Amiro, M. Aurela, A. G. Barr, T. A. Black, S. K. Carey, et al. 2020. The biophysical climate mitigation potential of boreal peatlands during the growing season. *Environmental Research Letters* 15: 104004.

[CR13] Hering, D., C. Schürings, F. Wenskus, K. Blackstock, A. Borja, S. Birk, C. Bullock, L. Carvalho, et al. 2023. Securing success for the nature restoration law. *Science* 382: 1248–1250. 38096279 10.1126/science.adk1658

[CR14] Hynynen, J., A. Ahtikoski, J. Siitonen, R. Sievänen, and J. Liski. 2005. Applying the MOTTI simulator to analyse the effects of alternative management schedules on timber and non-timber production. *Forest Ecology and Management* 207: 5–18.

[CR15] Hökkä, H., A. Ahtikoski, S. Sarkkola, and P. Väänänen. 2024a. Ash fertilization increases long-term timber production in drained nitrogen-poor Scots pine peatlands. *Canadian Journal of Forest Research* 54: 1142–1154.

[CR16] Hökkä, H., M. Palviainen, L. Stenberg, J. Heikkinen, and A. Laurén. 2024b. Changing role of water table and weather conditions in diameter growth of Scots pine on drained peatlands. *Canadian Journal of Forest Research*. 10.1139/cjfr-2024-0011.

[CR17] Jurasinski, G., A. Barthelmes, K. A. Byrne, B. H. Chojnicki, J. R. Christiansen, K. Decleer, C. Fritz, A. B. Günther, et al. 2024. Active afforestation of drained peatlands is not a viable option under the EU Nature Restoration Law. *Ambio* 53: 970–983. 10.1007/s13280-024-02016-5. 38696060 10.1007/s13280-024-02016-5 PMC11101405

[CR18] Juutinen, A., A. Tolvanen, M. Saarimaa, P. Ojanen, S. Sarkkola, A. Ahtikoski, S. Haikarainen, J. Karhu, et al. 2020. Cost-effective land-use options of drained peatlands–integrated biophysical-economic modeling approach. *Ecological Economics* 175: 106704.

[CR19] Kallio, A. M., and B. Solberg. 2018. Leakage of forest harvest changes in a small open economy: Case Norway. *Scandinavian Journal of Forest Research* 33: 502–510.

[CR20] Kellomäki, S. 2022. *Management of boreal forests: Theories and applications for ecosystem services*. Berlin: Springer.

[CR21] Korhonen, K. T., M. Räty, H. Haakana, J. Heikkinen, J.-P. Hotanen, M. Kuronen, and J. Pitkänen. 2024. Forests of Finland 2019–2023 and their development 1921–2023. *Silva Fennica* 58: 24045.

[CR22] Korkiakoski, M., P. Ojanen, J. P. Tuovinen, K. Minkkinen, O. Nevalainen, T. Penttilä, M. Aurela, T. Laurila, and A. Lohila. 2023. Partial cutting of a boreal nutrient-rich peatland forest causes radically less short-term on-site CO_2_ emissions than clear-cutting. *Agricultural and Forest Meteorology* 332: 109361.

[CR23] Koskinen, M., T. Tahvanainen, S. Sarkkola, M. W. Menberu, A. Laurén, T. Sallantaus, H. Marttila, A. K. Ronkanen, et al. 2017. Restoration of nutrient-rich forestry-drained peatlands poses a risk for high exports of dissolved organic carbon, nitrogen, and phosphorus. *Science of the Total Environment* 586: 858–869. 28215796 10.1016/j.scitotenv.2017.02.065

[CR24] Kulovesi, K., S. Oberthür, H. van Asselt, and A. Savaresi. 2024. The European climate law: Strengthening EU procedural climate governance? *Journal of Environmental Law* 36: 23–42.

[CR25] Laiho, R., S. Tuominen, S. Kojola, T. Penttilä, M. Saarinen, and A. Ihalainen. 2016. Heikkotuottoiset ojitetut suometsät—missä ja paljonko niitä on? *Metsätieteen Aikakauskirja* 2: 73–93 (in Finnish).

[CR26] Laine, A. M., M. Leppälä, O. Tarvainen, M. L. Päätalo, R. Seppänen, and A. Tolvanen. 2011. Restoration of managed pine fens: Effect on hydrology and vegetation. *Applied Vegetation Science* 14: 340–349.

[CR27] Laine, A., L. Mehtätalo, A. Tolvanen, S. Frolking, and E. S. Tuittila. 2019. Impacts of drainage, restoration and warming on boreal wetland greenhouse gas fluxes. *Science of the Total Environment* 647: 169–181. 30077847 10.1016/j.scitotenv.2018.07.390

[CR28] Laine, A.M., P. Ojanen, T. Lindroos, K. Koponen, L. Maanavilja, M. Lampela, J. Turunen, K. Minkkinen, et al. 2024. Climate change mitigation potential of restoration of boreal peatlands drained for forestry can be adjusted by site selection and restoration measures. *Restoration Ecology* 32: e14213.

[CR29] Laine, J., J. Silvola, K. Tolonen, J. Alm, H. Nykänen, H. Vasander, T. Sallantaus, I. Savolainen, et al. 1996. Effect of water-level drawdown on global climatic warming: Northern peatlands. *Ambio* 25: 179–184.

[CR30] Laine, J., Minkkinen, K., and Trettin, C. 2009. Direct Human Impacts on the Peatland Carbon Sink. In: Carbon Cycling in Northern Peatlands. *Geophysical Monograph Series*. American Geophysical Union. Eds. A. J. Baird, L. R. Belyea, X. Comas, A. S. Reeve, L. D. Slater, and D. Lee (Washington D.C.: AGU Geophysical Monograph Series) 184: 71–78.

[CR31] Lindner, M., J. B. Fitzgerald, N. E. Zimmermann, C. Reyer, S. Delzon, E. van Der Maaten, M. J. Schelhaas, P. Lasch, et al. 2014. Climate change and European forests: What do we know, what are the uncertainties, and what are the implications for forest management? *Journal of Environmental Management* 146: 69–83. 25156267 10.1016/j.jenvman.2014.07.030

[CR32] Lindroos, T.J. 2023. REFUGE4 – Radiative forcing calculation tool (4.1). Zenodo. 10.5281/zenodo.8304100

[CR33] Lohila, A., K. Minkkinen, J. Laine, I. Savolainen, J.P. Tuovinen, L. Korhonen, T. Laurila, H. Tietäväinen, et al. 2010. Forestation of boreal peatlands: Impacts of changing albedo and greenhouse gas fluxes on radiative forcing. *Journal of Geophysical Research: Biogeosciences*, 115.

[CR34] Lohila, A., K. Minkkinen, M. Aurela, J. P. Tuovinen, T. Penttilä, P. Ojanen, and T. Laurila. 2011. Greenhouse gas flux measurements in a forestry-drained peatland indicate a large carbon sink. *Biogeosciences* 8: 3203–3218.

[CR35] Makrickas, E., M. Manton, P. Angelstam, and M. Grygoruk. 2023. Trading wood for water and carbon in peatland forests? Rewetting is worth more than wood production. *Journal of Environmental Management* 341: 117952. 37196393 10.1016/j.jenvman.2023.117952

[CR36] Minkkinen, K. 1999. Effect of forestry drainage on the carbon balance and radiative forcing of peatlands in Finland (Doctoral dissertation, Helsingin yliopisto), https://helda.helsinki.fi/server/api/core/bitstreams/a299caeb-f57a-4794-8708-e9f6cc83365d/content

[CR100] Minkkinen, K., J. Laine, and H. Hökkä. 2001. Tree stand development and carbon sequestration in drained peatland stands in Finland – a simulation study. Silva Fennica 35(1): 55–69.

[CR37] Minkkinen, K., R. Korhonen, I. Savolainen, and J. Laine. 2002. Carbon balance and radiative forcing of Finnish peatlands 1900–2100—the impact of forestry drainage. *Global Change Biology* 8: 785–799.

[CR38] Minkkinen, K., P. Ojanen, M. Koskinen, and T. Penttilä. 2020. Nitrous oxide emissions of undrained, forestry-drained, and rewetted boreal peatlands. *Forest Ecology and Management* 478: 118494.

[CR39] Nieminen, M., H. Hökkä, R. Laiho, A. Juutinen, A. Ahtikoski, M. Pearson, S. Kojola, S. Sarkkola, et al. 2018. Could continuous cover forestry be an economically and environmentally feasible management option on drained boreal peatlands? *Forest Ecology and Management* 424: 78–84.

[CR40] Ojanen, P., K. Minkkinen, and T. Penttilä. 2013. The current greenhouse gas impact of forestry-drained boreal peatlands. *Forest Ecology and Management* 289: 201–208.

[CR41] Ojanen, P., A. Lehtonen, J. Heikkinen, T. Penttilä, and K. Minkkinen. 2014. Soil CO2 balance and its uncertainty in forestry-drained peatlands in Finland. *Forest Ecology and Management* 325: 60–73.

[CR42] Ojanen, P., and K. Minkkinen. 2019. The dependence of net soil CO_2_ emissions on water table depth in boreal peatlands drained for forestry. *Mires and Peat* 24: 27.

[CR43] Ojanen, P., and K. Minkkinen. 2020. Rewetting offers rapid climate benefits for tropical and agricultural peatlands but not for forestry-drained peatlands. *Global Biogeochemical Cycles* 34: e2019GB006503.

[CR44] Peichl, M., E. Martínez-García, J. E. Fransson, J. Wallerman, H. Laudon, T. Lundmark, and M. B. Nilsson. 2023. Landscape-variability of the carbon balance across managed boreal forests. *Global Change Biology* 29: 1119–1132. 36464908 10.1111/gcb.16534 PMC10108254

[CR45] Purre, A. H., T. Penttilä, P. Ojanen, K. Minkkinen, M. Aurela, A. Lohila, and M. Ilomets. 2019. Carbon dioxide fluxes and vegetation structure in rewetted and pristine peatlands in Finland and Estonia. *Boreal Environment Research* 24: 243–261.

[CR46] Sarkkola, S., H. Hökkä, H. Koivusalo, M. Nieminen, E. Ahti, J. Päivänen, and J. Laine. 2010. Role of tree stand evapotranspiration in maintaining satisfactory drainage conditions in drained peatlands. *Canadian Journal of Forest Research* 40: 1485–1496.

[CR47] Schwarze, R., J. O. Niles, and J. Olander. 2002. Understanding and managing leakage in forest–based greenhouse–gas–mitigation projects. *Philosophical Transactions of the Royal Society of London Series A: Mathematical, Physical and Engineering Sciences* 360: 1685–1703. 10.1098/rsta.2002.1040 12460492

[CR48] Tanttu, A. 1915. Tutkimuksia ojitettujen soiden metsittymisestä. Studien uber die Afforstungsfähigkeit der entwässerten Moore. *Acta Forestalia Fennica* 5: 1–211 (in Finnish with German summary).

[CR49] Tikkasalo, O. P., O. Peltola, P. Alekseychik, J. Heikkinen, S. Launiainen, A. Lehtonen, Q. Li, E. Martínez-García, et al. 2025. Eddy-covariance fluxes of CO2, CH4 and N2O in a drained peatland forest after clear-cutting. *Biogeosciences* 22: 1277–1300.

[CR50] Tong, C. H. M., K. D. Noumonvi, J. Ratcliffe, H. Laudon, J. Järveoja, A. Drott, M. B. Nilsson, and M. Peichl. 2024. A drained nutrient-poor peatland forest in boreal Sweden constitutes a net carbon sink after integrating terrestrial and aquatic fluxes. *Global Change Biology* 30: e17246. 38501699 10.1111/gcb.17246

[CR51] Turetsky, M.R., B.D. Amiro, E. Bosch, and J.S. Bhatti. 2004. Historical burn area in western Canadian peatlands and its relationship to fire weather indices. *Global Biogeochemical Cycles*, 18.

[CR52] Venäläinen, A., I. Lehtonen, M. Laapas, K. Ruosteenoja, O. P. Tikkanen, H. Viiri, V. P. Ikonen, and H. Peltola. 2020. Climate change induces multiple risks to boreal forests and forestry in Finland: A literature review. *Global Change Biology* 26: 4178–4196. 32449267 10.1111/gcb.15183 PMC7383623

[CR53] Wu, J., and N. T. Roulet. 2014. Climate change reduces the capacity of northern peatlands to absorb the atmospheric carbon dioxide: The different responses of bogs and fens. *Global Biogeochemical Cycles* 280: 1005–1024.

