# Supplementary material for: Rewetting drained boreal peatland forests does not mitigate climate warming in the twenty-first century
Source: Ambio. 2025 Aug 26;54(12):2107–17. doi: 10.1007/s13280-025-02225-6 (PMC12569233; doi:10.1007/s13280-025-02225-6)
Supplement: Supplementary file 1 — Supplementary file1 (PDF 1143 KB) [file 13280_2025_2225_MOESM1_ESM.pdf]

Supplementary material

Rewetting drained boreal peatland forests does not mitigate climate warming in the 21st century

Samuli Launiainen[1], Anssi Ahtikoski[2], Janne Rinne[1], Paavo Ojanen[3,5], Hannu Hökkä[4]

[1]Natural Resources Institute Finland, Bioeconomy & Environment, Ecosystems & Modeling, Latokartanonkaari 9, 00790 Helsinki, Finland

[2]Natural Resources Institute Finland, Natural Resources, Forest management, Tekniikankatu ,1 33720 Tampere, Finland

[3]Natural Resources Institute Finland, Natural Resources, Soil ecosystems, Latokartanonkaari 9, 00790 Helsinki, Finland

[4]Natural Resources Institute Finland, Natural Resources, Forest management, Paavo Havaksen tie 3, 90570 Oulu, Finland

[5]University of Helsinki, Department of Forest Sciences, Latokartanonkaari 7, 00790 Helsinki

Corresponding Author: samuli.launiainen@luke.fi

S0. Supplementary Figures

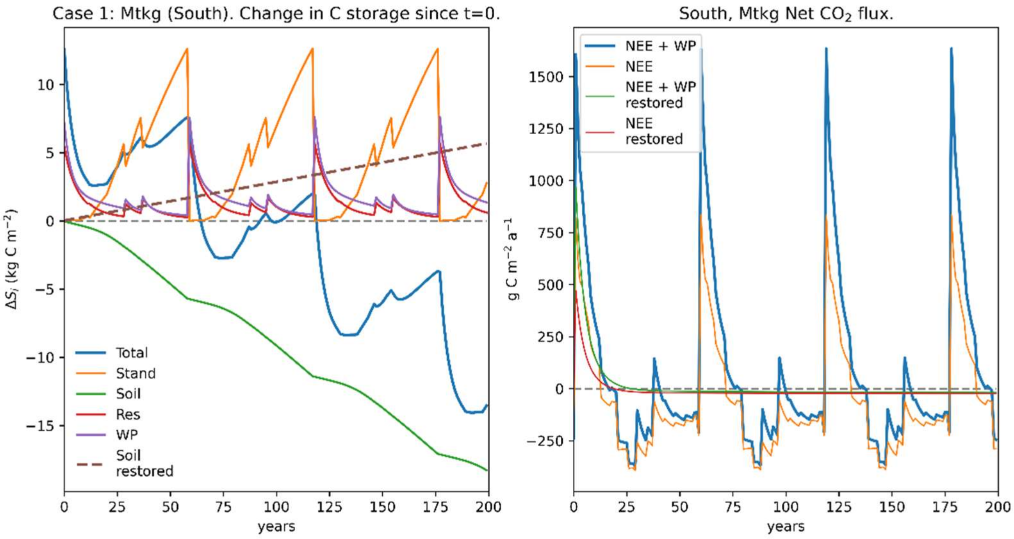

Fig. S1: Change of carbon (C) storages since *t*=0 in forestry scenario in the *Case 1*. The change of soil C storage in restoration scenario is shown for a reference (left). The annual net CO_2 flux "felt" by the atmosphere (positive values are net emissions, right). *Stand* = living vegetation, *Res* = harvest residues left at the site, *WP* = wood products. The net ecosystem exchange (NEE) includes net stand CO_2 uptake, soil CO_2 balance and CO_2emissions from decomposing harvest residues. NEE + WP includes also CO_2 emissions from wood products. For cutting residues and wood products the different pools are shown combined.

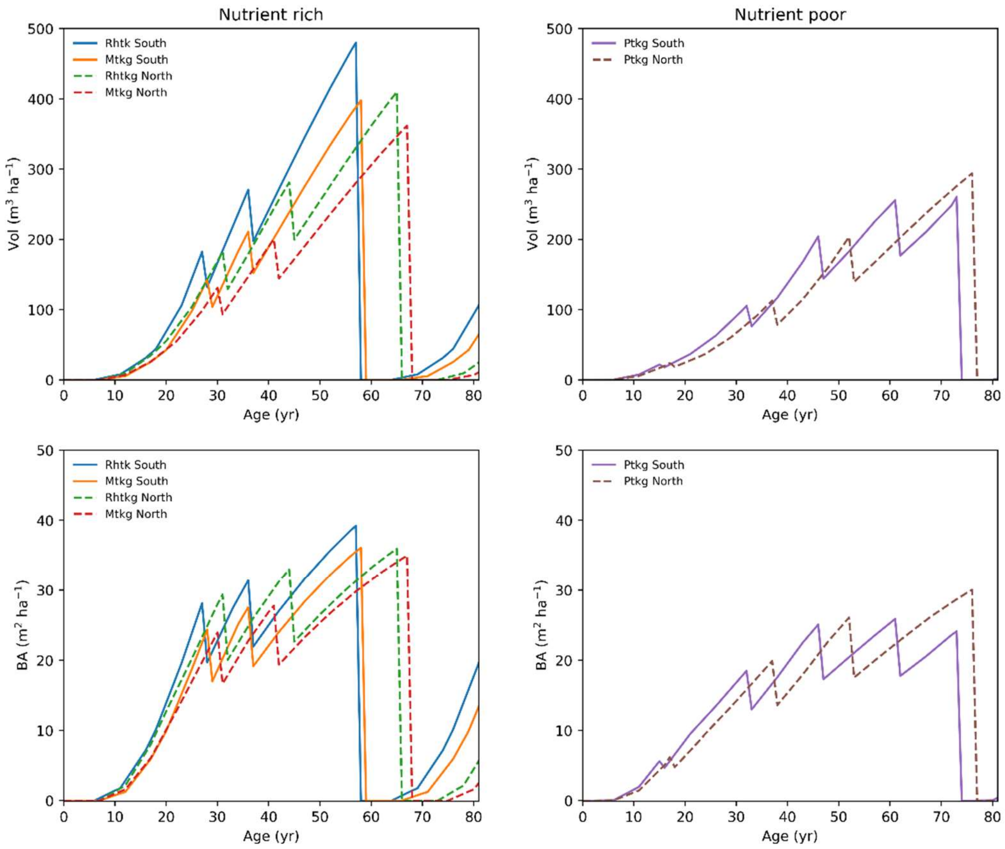

Fig. S2: Simulated development of stem volume (Vol) and basal area (BA) over a rotation cycle at nutrient-rich (FNR, herb-rich Rhtkg and mesotrophic Mtkg sites) and nutrient-poor (FNP, oligotrophic Ptkg site) drained peatland forests in Southern (Tampere) and Northern (Oulu) Finland. For details, see Sect. S1.5.

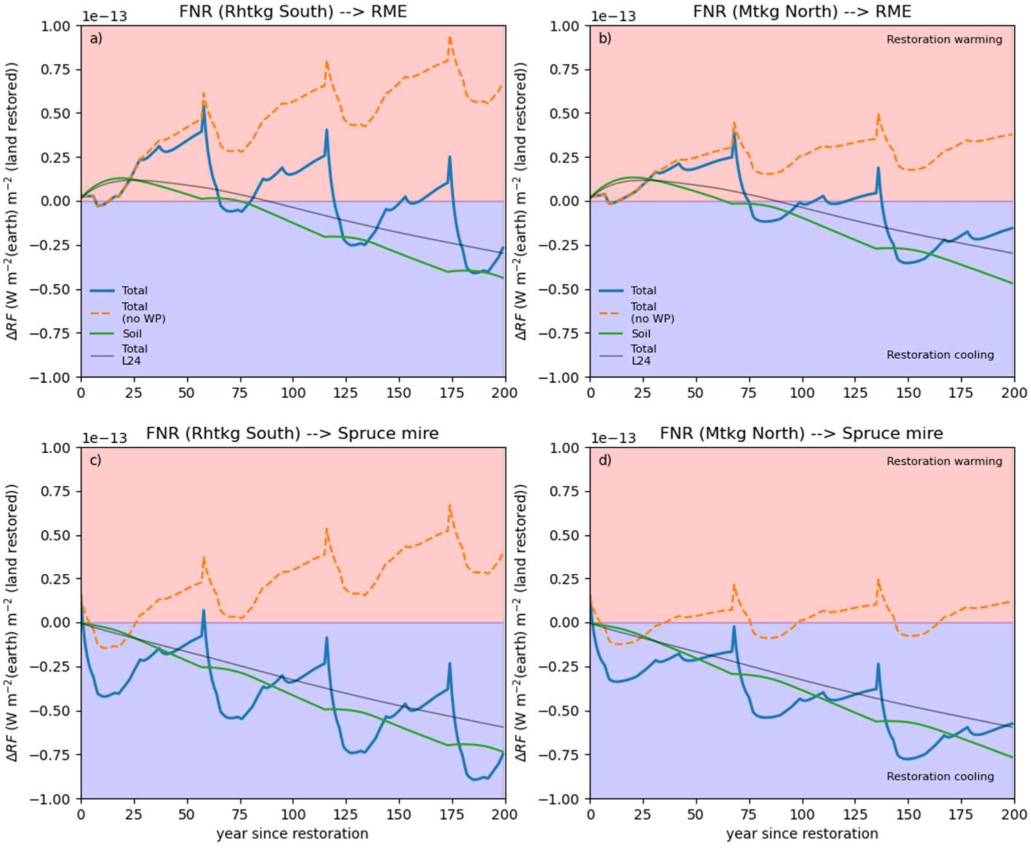

Fig. S3: As Fig. 2 but taking the forest stand dynamics (Fig. S2) from a more productive herb-rich (Rhtkg, Southern Finland; a,c) and from a less productive (Mtkg, Northern Finland; b, d) site. The warming impact of restoring to an open eutrophic/minerotrophic peatland (RME) is the stronger the higher productivity forests are restored.

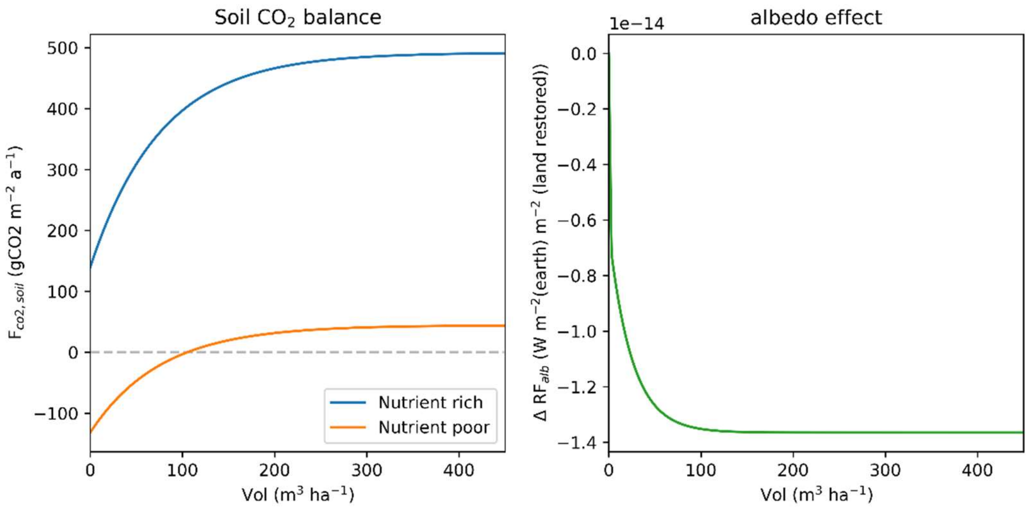

Fig. S4: Net soil CO_2 flux from drained peatland forests depends on stem volume (Vol., eq. S3; left). Approximate radiative forcing caused by albedo change (ΔRF_{alb}, eq. S14; restoration has a cooling effect) as a function of Vol. (right).

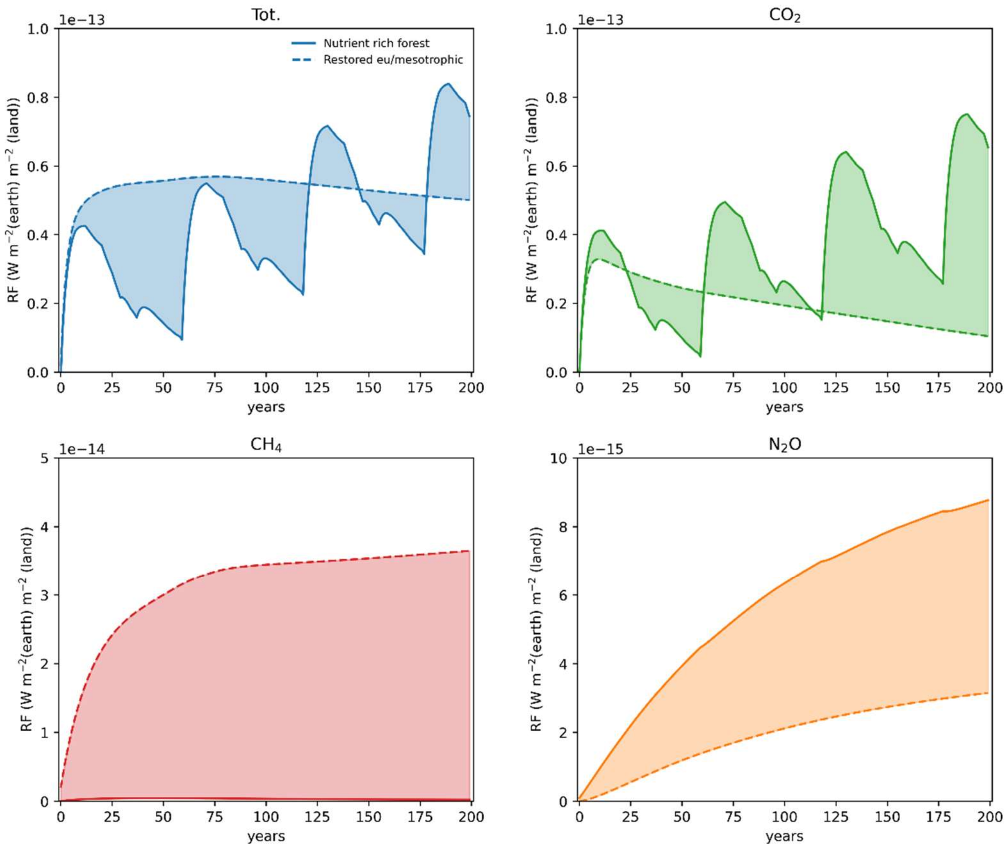

Fig. S5: Radiative forcing (RF) timeseries for a nutrient-rich forest (FNR, Mtkg in Southern Finland) and a restored open eutrophic/minerotrophic peatland (*Case 1*, Fig. 2a,b). Total radiative forcing from greenhouse-gases (GHGs) is the sum of individual GHGs' dynamic contributions (Sect. S1.3). The change in radiative forcing (ΔRF) due to restoration is the difference between restored and forestry drained peatland RF; i.e. restoration has a cooling effect when the dashed line is below the continuous line. Note that y-axis scale varies between panels.

S1. Detailed methods

S1.1 Carbon balance of drained and restored forest peatland

We build a simple bookkeeping model to track the change in carbon (C) storage ΔS_i (kg C m^{-2}) in a drained forest / restored peatland and harvested wood. Total change in the ecosystem and wood product C storage since the beginning of simulation period (*t*=0) is S_{tot}:

$$S_{tot}(t) = S_{soil}(t) + S_{tree}(t) + S_{res}(t) + S_{wps}(t) + S_{wpl}(t), \quad \text{(S1)}$$

where S_{soil} refers to soil C stock, S_{tree} that of tree stand, S_{wps} and S_{wpl} to short- and long-term wood products and S_{res} to harvest residues left on-site. We evaluate change in storages (ΔS_i) and resulting net CO_2 fluxes ($F_{c,i}$) to/from the atmosphere at annual timestep (Δt).

The ΔS_{soil} is the net soil CO_2 balance and varies with site fertility and water table depth (WT) according to Ojanen & Minkkinen (2019). Sarkkola et al. (2010, their Fig. 4) provide average summer WT as a function of stem volume (Vol, m^3 ha^{-1})

$$WT(Vol) = a + b \times c^{Vol}, \quad \text{(S2)}$$

where a=-50.56 cm, b=29.36 cm and c=0.9869 are parameters fitted to their Fig.4. Ojanen and Minkkinen (2019) showed that $F_{c,soil}$ (g CO_2 m^{-2} a^{-1}) depends linearly on WT, while the sensitivity differs between nutrient-rich (FNR) and nutrient-poor (FNP) stands. Combining their eq. 3&4 with eq. S2, the soil CO_2 balance becomes a function Vol., allowing it to vary during the stand rotation period as (Fig. S4):

$$F_{c,soil} = d_0 - d_1 b + d_1 b \times c^{Vol}, \quad \text{(S3)}$$

where parameters d_0 = -115 gCO_2 m^{-2} a^{-1} and d_1=12.0 gCO_2 m^{-2} a^{-1} cm^{-1} for FNR and -259 and 6.0 for FNP. Also Laine et al. (2024) estimated soil CO_2 balance based on Ojanen & Minkkinen (2019) but assumed a fixed WT and thus time-constant $F_{c,soil}$ over a stand rotation. For restored peatlands, we assume $F_{c,soil}$ varies across peatland types but is constant in time as in Laine et al. (2024, Table 1). These estimates are derived from observed peat accumulation rates in Turunen et al. (2002).

Tree stand C storage change $\Delta S_{tree} = F_{c,tree} - H$ is the balance between stand net biomass growth ($F_{c,tree}$) and periodical harvests (*H*). We estimate development of stem volume (Vol), basal area (BA and compartmental biomasses (BM), and the intensity and timing of harvests, for typical nutrient-rich (FNR) and nutrient-poor (FNP) sites and even-aged management (Sect. S1.5) using Motti forest simulator (Hynynen et al., 2005). $F_{c,tree}$ (g CO_2 m^{-2} a^{-1}) is based on the change of living tree C storage and equals the annual net CO_2 uptake from the atmosphere. For restored peatlands, we assume that annual vegetation net growth and thus change in vegetation CO_2 storage is negligible and $F_{c,tree}$=0.

The C in the harvested biomass is eventually released to the atmosphere as CO_2 from decomposition of harvest residues or burning of the wood products at the end of their life cycles. The annual change in these woody pools S_{wi} C storage is:

$$\Delta S_i(t) = \alpha_i H(t) - F(t)_{c,wi}, \quad \text{(S4)}$$

where α_i is the fraction of harvested biomass C allocated to pool *i* and $F_{c,wi}$ (g CO_2 m^{-2} a^{-1}) the annual release of C as CO_2 from pool *i* (Sect. S1.5). The conversion from units of C to that of CO_2 is by the ratio of molar masses. We assume wood products and residues release CO_2 to the atmosphere at a rate proportional to the pool size (1st order kinetics)

$$F_{c,wi}(t) = S_{wi}(t) e^{-1/\tau_i}, \quad \text{(S5)}$$

where τ_i (yr) is the mean lifetime of respective pools: 3 and 30 yr for short- and long-term wood products (Boiger et al., 2025), 3 yr for foliage and 7 yr for fine woody litter decomposing in aerobic conditions (Vavrova et al., 2009). For stumps and roots we assume $\tau = 30$ yr (Pearson et al., 2017), and an order of magnitude larger when they are decomposing in more anoxic conditions after rewetting.

The annual net flux of CO_2 (g CO_2 m^{-2} a^{-1}, negative values are uptake from the atmosphere) between the atmosphere and the peatland – wood product system is

$$F_{c,net}(t) = F_{c,soil}(t) - F_{c,tree}(t) + F_{c,res}(t) + F_{c,wp}(t), \quad \text{(S6)}$$

where the last two terms represent the total CO_2 release from harvest residues $F_{c,res} = \sum_{i=1}^{3} F_{c,res,i}$ and wood products $F_{c,wp} = \sum_{i=1}^{2} F_{c,wp,i}$, respectively. The change in $F_{c,net}$ and its components from drained (*d*) to restored (*r*) state

$$\Delta F_{c,i,d\rightarrow r} = F_{c,i,r} - F_{c,i,d} \tag{S7}$$

describes the impact of restoration on atmospheric CO_2 stock $S(t)_{c,atm}$ and causes negative (cooling) or positive (warming) change in the radiative forcing (ΔRF, W m^{-2} (earth) m^{-2} (land restored)) (Sect. S1.3). The component CO_2 fluxes (eq. S6), their changes and resulting ΔRFs are additive, which enables analyzing their separate and joint effect on the climate impact of restoration.

S1.2 CH_4 and N_2O balance of drained and restored forest peatland

Great majority of peatland methane (CH_4) and nitrous oxide (N_2O) source/sink processes take place in soils and vegetation at the soil surface (Ojanen and Minkkinen, 2019). Thus, we follow Laine et al. (2024) and assume annual net CH_4 and N_2O flux between the peatland and the atmosphere equal their net soil fluxes, i.e. $F_{ch4,net} = F_{ch4,soil}$, and use peatland-type specific annual values from Laine et al. (2024, Table 1). The CH_4 and N_2O fluxes are based on literature survey of chamber-measured fluxes in Finland and similar conditions in the boreal zone; see Laine et al. (2024) for details.

S1.3 Radiative forcing from CO_2, CH_4 and N_2O

Changes in the net uptake/emission of CO_2, CH_4 and N_2O ($\Delta F_{k,d\rightarrow r}(t)$) due to restoration affects their atmospheric stocks, and alter the radiative forcing ΔRF_k (eq. 2, W m^{-2} (earth) m^{-2} (land restored)) contributing either to warming ($\Delta RF_k > 0$) or cooling ($\Delta RF_k < 0$). A pulse of gas *k* emitted to the atmosphere at time t_0 creates a dynamic change in $\Delta S_{a,k}(t)$, that for CH_4 and N_2O is represented as

$$\Delta S_{a,k}(t) = \Delta S_{a,k}(t_0) \times e^{-t/\tau_k}, \tag{S8}$$

where τ_k is the mean atmospheric lifetime of gas *k* (12 yr for CH_4 and 109 yr for N_2O). Emitted CO_2 pulse can undergo alternative decay pathways *j* in the atmosphere, leading to:

$$\Delta S_{a,co2}(t) = \Delta S_{a,co2}(t_0) \times \left[\beta_0 + \textstyle\sum_{j=1}^{3} \beta_j e^{-t/\tau_{co2,j}}\right], \tag{S9}$$

where β_j (-) is the fractional contribution of each decay pathway, which respective $\tau_{co2,j}$ ranges from 4.3 to 394 yr (Frolking et al., 2006; Lindroos, 2023). Note that ca. 22% (β_0) of the emitted CO_2 pulse accumulates in the atmosphere explaining why reaching global CO_2 neutrality is not sufficient to reduce atmospheric CO_2 concentrations. The atmospheric lifetime functions (eq. S8&9) describe the dynamical response of atmospheric GHG stocks to net surface emission/sink (or their difference between restored and drained peatland, e.g. $\Delta F_{co2,d\rightarrow r}(t)$), accounting for the aggregated effects of atmospheric chemistry and land-ocean GHG sinks.

After restoring a peatland at year *t*=0, the cumulated change in atmospheric stock of gas *k* at year *t* is thus the integral of each annual pulse emission/removal (Frolking et al., 2006)

$$\Delta S_{a,co2}(t) = \int_{t=0}^{t} \Delta F_{k,d\rightarrow r}(t')\ e^{(t'-t)/\tau_k}\, dt', \tag{S10}$$

where $\Delta F_{k,d\rightarrow r}(t')$ is the restoration impact on net emissions/sinks of gas *k* on year *t'*. There are different ways to compute the change in radiative forcing $\Delta RF_k(t)$ from $\Delta S_{a,k}(t)$. For instance, Frolking et al. (2006) uses a simple and intuitive approach

$$\Delta RF_k(t) = \xi_k E_k \times \Delta S_{a,k}(t), \tag{S11}$$

where E_k(W m^{-2} (earth) kg^{-1} (gas)) is a constant radiative efficiency of gas *k*, and ξ_k (-) a multiplier for indirect effects (Ramaswamy et al., 2001). So that our results are directly comparable to those of Laine et al. (2024), we adopt the same REFUGE 4 method (Lindroos, 2023) to compute mean annual radiative forcing $\Delta RF_k(t)$.

The radiative forcings from different gases and (eco)system components are additive, and the time-dependent change of the total radiative forcing caused by forest peatland restoration can be written as:

$$\Delta RF_{tot}(t) = \Delta RF_{co2,soil}(t) + \Delta RF_{tree}(t) + \Delta RF_{res}(t) + \Delta RF_{wp}(t) + \Delta RF_{ch}\ (t) + \Delta RF_{n2o}(t) + \Delta RF_{alb}(t), \quad \text{(S12)}$$

where the last term ΔRF_{alb} approximates the decreased radiative forcing caused by increased surface albedo when a forest peatland is restored to an open peatland (Suppl. S1.4).

S1.4 Radiative forcing from surface albedo change

Restoration increases peatland reflectivity (surface albedo increases, $\Delta\alpha$) and thus smaller fraction of incoming global radiation ($SW_{\downarrow}$) is absorbed compared to a mature forest. The global radiative forcing caused by $\Delta\alpha$ can be approximated by (Sieber et al., 2019)

$$\Delta RF_{alb} = SW_{\downarrow} \times \Delta\alpha \times \tau_{atm} \times \frac{A}{A_{earth}}, \quad \text{(S13)}$$

where $SW_{\downarrow}$ is the incoming global radiation at the surface (W m^{-2}), A = 1 m^2 and A_{earth} = 5.1e14 m^2 is the surface area of the Earth, and τ_{atm} (-) the mean SW transmissivity of the atmosphere. All three first terms in eq. S13 have a seasonal cycle, and $\Delta\alpha$ (forest → peatland) depends on the forest structure, being negligible in young stands and saturating in mature stands when the canopy closes. For simplicity, we approximate annual ΔRF_{alb} using results of Lohila et al. (2010), who measured albedos of forested and open boreal peatlands and showed how ΔRF_{alb} caused by draining an open peatland for forestry varies over stand rotation in FNR and FNP forests in Finland. We digitized their Fig. 2 and 7 (FNR in Southern Finland), converted the stand C storage to stand volume using biomass expansion factors (Lehtonen et al., 2004), and fitted a non-linear function to predict the ΔRF_{alb} from Vol as (Fig. S4):

$$\Delta RF_{alb}(Vol) = a_0 \times (1 - be^{-cV}\ ), \quad \text{(S14)}$$

where b=0.53and c=0.04 are fitting parameters, and $a_0 = SW_{\downarrow} \times \Delta\alpha \times \tau_{atm} \times \frac{A}{A_{earth}}$ (W m^{-2} (earth) m^{-2} (land restored)) is the ΔRF_{alb} when $Vol \to \infty$ (i.e. the radiative forcing when a mature forest peatland is converted to an open peatland). Based on Lohila et al. (2010), a_0= -1.0e-14 W m^{-2} (earth) m^{-2} (land restored) in Southern Finland. Recently, Peräkylä et al. (2025) compared albedos and reported the difference in annually absorbed short-wave radiation (i.e. $SW_{\downarrow} \times \Delta\alpha$, their Fig. 8) at adjacent open peatland and mature forests in Southern and Northern Finland. Using average τ_{atm}=0.65 from Lohila et al. (2010), their results yield a_0= -1.36e-14 (used in this work) and a_0= -1.56e-14 W m^{-2} (earth) m^{-2} (land restored) in Southern and Northern Finland, respectively. The larger cooling effect in Northern Finland is due to later snow melt, as during the winter snow-cover the albedo of open peatland is much larger than that of forest, while the difference is nearly negligible in summer (Lohila et al., 2010; Peräkylä et al., 2024).

S1.5 Tree stand C storage and allocation of harvested biomass C into wood products and harvest residues

Dynamics of tree stand C storage $S_{tree}(t)$ (g C m-2) is predicted using the Motti forest simulator (Hynynen et al., 2005; Fig. S1&2). Motti computes stand dynamics using linked semi-empirical models to describe the effect of species composition, tree age and size, between-tree competition, site fertility, climatic conditions, and management measures on tree growth and mortality. Stand level models are applied for predicting natural regeneration and early growth of young seedling stands until their dominant height reaches 7 m (Hynynen et al. 2014). Size distribution models (Siiplehto 2006; Siipilehto & Mehtätalo 2013) are then applied to produce tree-lists, after which individual-tree models are used

for predicting growth (Hynynen et al. 2002). Tree level models have been calibrated against long-term growth experiments and National Forest Inventory (NFI) measurements, and model predictions compare well with observed stand dynamics. In Finland, rotation period on drained peatlands is 50–100 yr depending on site type, climate and management goals, and contains 1–3 thinnings and a final clear-cut.

We predicted the development of stand attributes and harvest dynamics (e.g. Fig. S1) for FNR and FNP forests as follows: Starting from stand regeneration by planting or seeding, we simulated stand development (e.g. stem volume and basal area growth, biomass components) until final felling. Regeneration and management measures such as thinning's and ditch network maintenance were done following current practices in peatland forestry in Finland (Vanhatalo et al. 2019). For FNR, we consider spruce on eutrophic herb-rich Rhtkg and mesotrophic/Vaccinium myrtillus type Mtkg site types, while FNP forests are represented by pine on oligotrophic/Vaccinium vitis-idaea type Ptkg type. To cover climatic variability, we simulate growth rates and $S_{tree}(t)$ both for Southern Finland (Tampere, temperature sum 1226 dd°C, base temperature +5°C) and Northern Finland (Oulu, 1086 dd°C). In Fig. 2–3, we use mesotrophic Mtkg stand in Southern Finland as case-example, while the effect of growth and $S_{tree}(t)$ variability due to site-type and climate is accounted for in Fig. 4 and Fig. S3.

We compute S_{tree} from the simulated total above- and below ground dry biomass assuming that half of dry biomass is C. The $F_{c,tree}$ does not include ground vegetation and therefore likely underestimates vegetation net primary production (NPP) during early stages of stand development (e.g. Peichl et al., 2023). When stand is harvested, we assume the harvested biomass C is transferred into long- or short-term wood products and harvest residues left to the site (Fig. S1). Harvested stem biomass is allocated to long-term wood products (timber, plywood, mean lifetime τ = 30 yr) based on the sawlog fraction (f_{log}, available from Motti simulations) of total stem C multiplied by the saw yield (α = 0.4). The remaining part of stem biomass moves into short-term wood product pool (e.g. fibre, paper, cardboard, bioenergy, τ = 3 yr). The mean lifetimes are based on Boiger et al. (2025).

Fig. S1 illustrates C storage dynamics and net fluxes to the atmosphere when wood products are included/excluded from the calculations. Example is FNR stand (mesotrophic Mtkg) in Southern Finland, with total wood production of 525 m^3 over the 58 yr rotation. In this scenario, 28% of the harvested stem wood C was allocated into long-term wood products over the rotation period. Fig. S2 shows the development of key stand attributes in the same simulation.

S1.6 Restoration pathways

Laine et al. (2024) divided drained forest peatlands into two classes, which is followed here: The nutrient-rich forests (FNR) include eutrophic herb-rich (Rhtkg) and mesotrophic/Vaccinium myrtillus type (Mtkg) site types. Before drainage for forestry, they have been forested or sparsely forested spruce or pine mires with admixture of pubescent birch. Currently they are dominated by Norway spruce and belong to the most productive forests in Finland; the mean annual increment in late rotation stand varies from 8 to 11 in Southern Finland and from 6 to 8 m^3 ha^{-1} a^{-1} in Northern Finland (National Forest Inventory; Korhonen et al., 2024). These net growth rates correspond to net CO_2 uptake ($F_{c,tree}$) of 1050–1450 g CO_2 m^{-2} a^{-1}. Among the nutrient-poor (FNP) drained forest peatlands, oligotrophic/Vaccinium vitis-idaea type (Ptkg) is particularly important for wood production. At FNP sites, Scots pine is the dominant species with corresponding late-rotation mean annual increment from 4 to 5 m^3 ha^{-1} a^{-1} ($F_{c,tree}$ 500–750 gCO_2 m^{-2} a^{-1}). FNP has its origin in nutrient-poor bogs or poor oligotrophic pine fens.

We explore following restoration pathways: FNR stands can become tree-covered spruce mires, open eutrophic/mesotrophic or oligotrophic mires. FNP stands can be restored to pine mires or open oligotrophic mires.

Supplementary References

Boiger, T., Mair-Bauernfeind, C., Asada, R. and Stern, T. 2025. Optimizing the utilization of harvested wood products for maximum greenhouse gas emission reduction in a bioeconomy: A multi-objective optimization approach. *Journal of Environmental Management*, 373, 123424.

Haapanen, M., Hynynen, J., Ruotsalainen, S., Siipilehto, J., and Kilpeläinen, M-L. 2016. Realised and projected gains in growth, quality and simulated yield of genetically improved Scots pine in southern Finland. *European Journal of Forest Research* 135, 997–1009 (2016). https://doi.org/10.1007/s10342-016-0989-0

Hynynen, J., Ahtikoski, A., Siitonen, J., Sievänen, R. and Liski, J. 2005. Applying the MOTTI simulator to analyse the effects of alternative management schedules on timber and non-timber production. *Forest Ecology and Management*, 207(1-2), 5–18.

Korhonen, K.T., Räty, M., Haakana, H., Heikkinen, J., Hotanen, J.P., Kuronen, M. and Pitkänen, J., 2024. Forests of Finland 2019-2023 and their development 1921-2023. *Silva Fennica*, vol. 58 no. 5 article id 24045.

Laine, A.M., Ojanen, P., Lindroos, T., Koponen, K., Maanavilja, L., Lampela, M., Turunen, J., Minkkinen, K. and Tolvanen, A. 2024. Climate change mitigation potential of restoration of boreal peatlands drained for forestry can be adjusted by site selection and restoration measures. *Restoration Ecology*, 32(7), p.e14213.

Lehtonen, A., Mäkipää, R., Heikkinen, J., Sievänen, R. and Liski, J. 2004. Biomass expansion factors (BEFs) for Scots pine, Norway spruce and birch according to stand age for boreal forests. *Forest Ecology and Management*, 188(1–3), 211–224.

Lohila, A., Minkkinen, K., Laine, J., Savolainen, I., Tuovinen, J.P., Korhonen, L., Laurila, T., Tietäväinen, H. and Laaksonen, A. 2010. Forestation of boreal peatlands: Impacts of changing albedo and greenhouse gas fluxes on radiative forcing. *Journal of Geophysical Research: Biogeosciences*, 115(G4).

Ojanen, P. and Minkkinen, K. 2019. The dependence of net soil CO2 emissions on water table depth in boreal peatlands drained for forestry. *Mires and Peat*, 24(Article 27), p.27.

Pearson, M., Laiho, R. and Penttilä, T. 2017. Decay of Scots pine coarse woody debris in boreal peatland forests: mass loss and nutrient dynamics. *Forest Ecology and Management*, 401, 304–318.

Peichl, M., Martínez-García, E., Fransson, J.E., Wallerman, J., Laudon, H., Lundmark, T. and Nilsson, M.B. 2023. Landscape-variability of the carbon balance across managed boreal forests. *Global Change Biology*, 29(4), 1119–1132.

Peräkylä, O., Rinne, E., Ezhova, E., Lintunen, A., Lohila, A., Aalto, J., Aurela, M., Kolari, P. and Kulmala, M. 2025. Comparison of shortwave radiation dynamics between boreal forest and open peatland pairs in southern and northern Finland. *Biogeosciences*, 22(1), 153–179.

Sieber, P., Ericsson, N. and Hansson, P.A. 2019. Climate impact of surface albedo change in Life Cycle Assessment: Implications of site and time dependence. *Environmental Impact Assessment Review*, 77, 191–200.

Siipilehto, J. 2006. Linear prediction application for modelling the relationships between a large number of stand characteristics of Norway spruce stands. *Silva Fennica* 40(3):517–530.

Siipilehto, J. & Mehtätalo, L. 2013. Parameter recovery vs. parameter prediction for the Weibull distribution validated for Scots pine stands in Finland. *Silva Fennica* 47(4):1–22.

Turunen, J., Tolonen, K., Tomppo, E., and Reinikainen, A. 2002. Estimating carbon accumulation rates of undrained mires in Finland – application to boreal and subarctic regions. *The Holocene* 12:69–80.

Vanhatalo, K., Väisänen, P., Joensuu, S., Sved, J., Koistinen, A. and Äijälä, O. 2019. Metsänhoidon suositukset suometsien hoitoon, työopas. Tapion julkaisuja. ISBN 978-952-5632-74-3. 119 p. (In Finnish)

Vávřová, P., Penttilä, T. and Laiho, R. 2009. Decomposition of Scots pine fine woody debris in boreal conditions: Implications for estimating carbon pools and fluxes. *Forest Ecology and Management*, 257(2), 401–412.
